# Identifying the Key Risk Factors of Mega Infrastructure Projects from an Extended Sustainable Development Perspective

**DOI:** 10.3390/ijerph18147515

**Published:** 2021-07-14

**Authors:** Yuanli Li, Pengcheng Xiang, Kairui You, Jin Guo, Zhaowen Liu, Hong Ren

**Affiliations:** 1 School of Management Science and Real Estate, Chongqing University, Chongqing 400045, China; liyuanli@cqu.edu.cn (Y.L.); youkairui@cqu.edu.cn (K.Y.); guojeen@cqu.edu.cn (J.G.); renhong@cqu.edu.cn (H.R.); 2 International Research Center for Sustainable Built Environment, Chongqing University, Chongqing 400045, China; 3 Construction Economics and Management Research Center, Chongqing University, Chongqing 400045, China; 4 Faculty of Civil Engineering and Geosciences, Delft University of Technology, Stevinweg 1, 2628 CN Delft, The Netherlands; z.liu-8@tudelft.nl

**Keywords:** mega infrastructure projects, sustainable development, risk identification, fuzzy set theory, coordination

## Abstract

Mega infrastructure projects (MIPs) have become increasingly important to the realization of sustainable development in China. Sustainable development is a process of dynamic balance, and coordinating the triple bottom line (the environmental, social, and economic dimensions) will enable more sustainable development of MIPs. However, previous studies have lacked consideration of coordination when applying sustainable development principles to the systematic identification of risks to MIPs. The goals of this study were to clarify the definition and dimensions of the sustainable development of MIPs and to identify the key risks of MIPs. A literature review was performed to extend the definition of sustainable development of MIPs by combining the triple bottom line with a fourth coordination dimension. A conceptual model of MIP risk identification was then proposed from an extended sustainable development perspective, 22 sustainability elements and 75 risk factors were identified, and the key risk factors were determined based on the interview responses and fuzzy set theory. The results show that economic risks have a high probability, social risks have a high loss, environmental risks have an intermediate probability and loss, and coordination risks have the greatest impact. In addition, the three most important key risk factors were found to be construction and installation cost overruns, land acquisition and resettling cost overruns, and information sharing with the public. Identifying key risk factors can provide information to help stakeholders understand the risk factors associated with MIPs and formulate reasonable risk response strategies.

## 1. Introduction

Mega infrastructure projects (MIPs) are large-scale engineering facilities such as transportation systems, water supply systems, energy systems, or communication systems that provide basic public services for social production, economic development, and residents’ livelihoods [1]. At least three features associated with MIPs are notable [2]: (1) MIPs are costly and require high amounts of labor, physical and financial resources, and the total amount of project funding usually exceeds many billions. (2) MIPs are strategic and public welfare, which generally are key projects in the national or local government’s economic development plan, and usually are commissioned by the government and delivered by competent private contractors and suppliers. (3) MIPs have long-term impacts on the national or regional economy, civil society, and natural environment, even affecting multiple generations in the long term. As MIPs are the foundation of social and economic development, investing in the construction of such projects is particularly important in developing countries. Taking China as an example, many MIPs have been constructed in order to stimulate the national economy, including the Three Gorges Dam, Hong Kong–Zhuhai–Macao Bridge, and Sichuan–Tibet Railway. Compared with small- and medium-scale infrastructure projects, MIPs are distinguished by their considerable investment, numerous stakeholders, major political or external influences, and long life cycles. Combined, these characteristics mean that the risks are more complex and have a greater impact on MIPs with increasing project scale and complexity. Therefore, the success of an MIP depends on effective risk management [3].

Risk identification is considered the most important stage of risk management, because a risk cannot be managed until it has been identified [4]. Many scholars have accordingly made significant efforts to identify and analyze the risk factors of MIPs and extend the scope of risk recognition to include scope changes, time delays, cost overruns, quality issues, security incidents, and environmental safeguards [5,6,7,8,9]. However, it has often been reported that many negative impacts and risks remain associated with MIPs that can result in deleterious events and outcomes. For example, in China, the mass migration associated with the Three Gorges Dam has led to the marginalization of some populations [10], and the Hong Kong–Zhuhai–Macao Bridge has created ecological problems as it crosses the Pearl River Estuary Chinese White Dolphin National Nature Reserve [11]. Furthermore, although the Beijing–Shanghai high-speed railway project promotes regional economic development and provides convenient transportation infrastructure, the planning and location decision-making process did not consider the impact of the project on the health of the environment or residents. As a result, the project has been opposed by residents along its planned route, leading to repeated safety reviews, route changes, and rejection by the Ministry of Environmental Protection [12]. There are many similar problems associated with various other MIPs around the world.

Indeed, although MIPs can provide many economic benefits, they can also have negative impacts, especially in social and environmental terms [13,14]. However, existing research into risk identification has, to date, primarily considered cost, time, and quality as targets (the “iron triangle” principle), thereby paying more attention to the MIPs themselves. This singular focus has led to doubts regarding the effectiveness and adequacy of current MIP risk identification. Furthermore, with the increase in the sustainable development awareness of the general population, the impact of MIPs on sustainable development has become a focus of attention in academia, introducing a wide range of new risks to be considered. Therefore, MIP risk identification must be improved by incorporating the attributes embodied in sustainable development principles.

Sustainable development principles are widely described using the triple bottom line (TBL) theory. The TBL originated from the well-known Brundtland report [15], which considers the economic, social, and environmental dimensions of a project [16]. The relative importance of these three pillars in TBL theory has led to an increasingly fierce argument over what constitutes sustainable development of infrastructure, resulting in multiple interpretations, ranging from priority pillars to compatibility of the three pillars and expansion of other dimensions. For example, Cole suggested that if the three pillars are incompatible, there will always be the chance that development will prioritize one sustainability pillar over another to suit a particular agenda [17]. Initially, the primary “agenda” associated with infrastructure development was the general meeting of social and economic needs; remaining within absolute global environmental limits has tended to be a secondary consideration. However, Parkin [18] and Raworth [19] proposed the “nested” view of sustainable development, demonstrating the importance of remaining within absolute environmental limits when meeting social and economic needs. Giddings et al. [20] believed that the three pillars are equally important as different but complementary dimensions of sustainable development. In addition, some studies have reasonably criticized the three-pillar model as sustainable development is a process of dynamic balance [21]. Kemp et al. [22] argue that simply adding the indicators of the three dimensions of sustainability ignores the interconnection and dynamic effects of social, environmental, and economic perspectives. Devolder and Block [23] also emphasized the importance of synergy between dimensions. Kivilä et al. [24] identified the dependencies between the environmental, social, and economic dimensions of sustainability, and pointed out that the sustainable project management of infrastructure delivery projects should involve and build on stakeholder cooperation, include life cycle thinking, and balance the three dimensions of sustainability. The addition of topics related to management actions in the framework of infrastructure sustainability assessment helps to balance the relevance of the three pillars of sustainable development [25]. Therefore, some studies have proposed other dimensions that go beyond the three pillars of social, ecological, and economic sustainability. Hueskes et al. [26] developed the infrastructure sustainability assessment framework in addition to the three-pillar to involve transformative changes and political system dimensions. Liu et al. [27] integrated managerial infrastructure sustainability (IS) with the triple bottom line in the infrastructure IS metric system. In addition, several international organizations have published regional IS rating systems, including the IS rating program initiated by the Infrastructure Sustainability Council of Australia [28], the Envision system organized by the Institute for Sustainable Infrastructure [29], and the institutional dimension proposed by the Inter-American Development Bank [30]. All these systems consider additional dimensions such as balance, management, or other intangibles that complement the three pillars of sustainable development. It can be concluded from these research works that the management level has become the fourth pillar to support TBL’s assessment of the sustainable development of infrastructure.

However, research on risk management of the sustainable development of MIPs rarely considers the management level. MIP is a complex system, and its risks present multi-dimensional complexity and interaction [31]. If the unbalanced integration of the three dimensions leads to greater MIP risks, it is difficult to achieve sustainable development. For example, mega hydropower infrastructure projects have reduced energy consumption and brought huge economic benefits, but some dam projects have caused serious environmental problems and ecological disasters. With the intensification of environmental problems, incidents of confrontation among the public, enterprises, and the government have further exacerbated. Economic benefits, environmental issues, and social disputes can no more be handled separately. To achieve sustainable development, all three dimensions must be balanced. Coordination management emphasizes the comprehensive use of various management methods in decision-making, organization, management, and MIP responsibilities to coordinate the relationship between stakeholders, and unify their motivations, goals, attitudes, and actions. When all stakeholders of the project organization work together toward a common goal, several unnecessary disputes and quarrels can be avoided, which synergistically promotes the sustainability of the project in all three dimensions [24]. Therefore, when conducting risk management research on the sustainable development of MIPs, it is crucial to incorporate the coordination dimension to examine the potential risks that may cause imbalance of the three pillars.

Several studies have attempted to identify MIP risk factors from a limited sustainable development perspective. Yuan et al. [32] identified the social risks of Chinese transportation MIPs from a sustainable development perspective using a literature review, and emphasized that the impact of social and environmental change on sustainability may lead to increasing social risks. Shi et al. [33] used expert meetings and interviews to analyze a water supply MIP in China and identified 12 critical social risk factors related to the project in terms of its legality and rationality, land acquisition and housing demolition, and construction phase. Song et al. [34] used case studies to identify 10 critical environmental risks affecting public–private partnership (PPP)-based MIPs, including government decision-making, government credit, legal and policy, and technical risks. Using a literature review, Bai et al. [35] identified the risks affecting the sustainable development of PPP-based MIPs in China from five perspectives: culture and society, cost and economy, ecology and environment, project and organization, and politics and policy. They asserted that the sustainability risks of different projects were different. However, most of these studies tended to narrowly define risk factors, only considering a single dimension of a sustainable development perspective and thereby leading to fragmented risk identification. Thus, current studies have failed to systematically identify risks in the multiple dimensions embodied in sustainable development principles. In addition, while identifying the risks, limited studies have considered the balance integration of all the three dimensions of the TBL theory, or the ability to meet the diverse needs of multiple stakeholders in the MIP. Therefore, several critical gaps remain in the research into MIP risk identification.

These gaps motivated us to incorporate a “coordination dimension” to the TBL that extends the sustainable development of the MIP, and systematically identifies the MIP key risks based on four dimensions. The results of this research are intended to help decision makers who invest in and make decisions governing MIPs to understand the associated risk factors by providing them with valuable information, thereby improving the sustainability of MIPs.

The remainder of this paper is structured as follows: Section 2 clarifies the study framework, explains the main study method employed in each stage; the key MIP risk factors are identified and discussed in Section 3; and Section 4 summarizes the conclusions, contributions of the study, and future directions for research.

## 2. Methods

**Figure 1:**
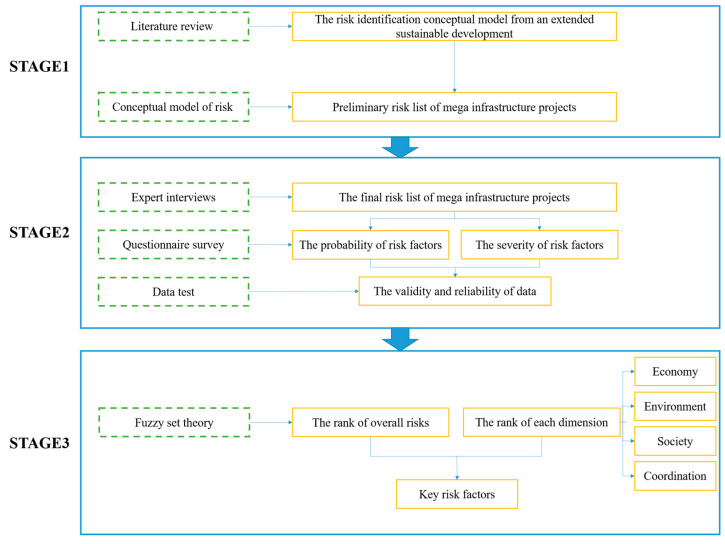
The research framework.

A hybrid research method was employed in this study, the research framework is presented in Figure 1. First, based on a review of previous research results, a risk identification conceptual model that integrates economic, social, environment and coordination dimension is proposed. In addition, the potential risk of MIPs are identified through the proposed conceptual model. Then, expert interviews and questionnaires was conducted to collect data. Based on the data collected, the key risk factors were screened using fuzzy set theory. Finally, associating with key risk factors, we proposed the corresponding methods to reduce the risks of MIPs from an extended sustainable development perspective, so as to enhance MIPs to achieve sustainable development.

### 2.1. Stage 1

#### 2.1.1. Proposed Conceptual Model

**Figure 2:**
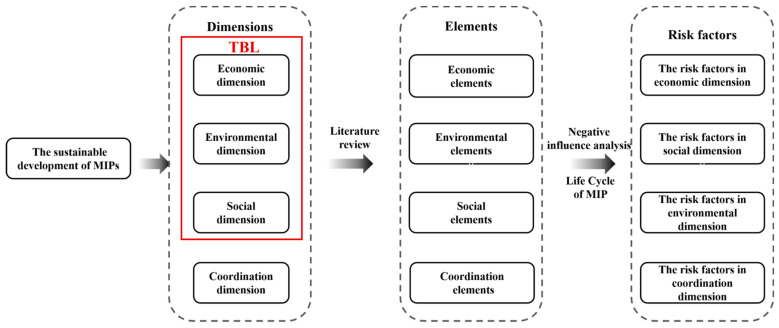
The risk identification conceptual model. Note: MIPs: Mega Infrastructure Projects; TBL: Triple Bottom Line.

The purpose of risk identification is to identify, judge, and classify the potential risk factors that a project may face in the process of achieving its goals [4]. A comprehensive review of the goals of MIPs from a sustainable development perspective is a prerequisite for clearly identifying their risk factors. Therefore, according to the principle of sustainable development of MIPs with four dimensions after being extended, this study proposes a risk identification conceptual model as follows: (1) review the sustainability dimensions in the implementation process of MIPs through literature analysis and subdivided into sustainable development elements; (2) within the lifecycle of MIPs, identify various risk factors that may cause MIPs to have a negative impact on each sustainable development element. The risk identification conceptual model is shown in Figure 2.

#### 2.1.2. Literature Research

The formulation and clarification of goals can be accomplished through indicator elements, which can provide more meaningful information to decision makers and other stakeholders, especially in the complex decision-making systems of MIP. In this study, the elements under the four sustainable development dimensions of economy, society, environment, and coordination were reviewed based on a literature analysis. We searched for articles using keywords from the Web of Science. Keywords included “mega infrastructure sustainability,” “mega infrastructure sustainability assessment,” and “mega infrastructure sustainability indicators.” The selection of the literature was guided by two principles: (1) the use of publications focused on MIP sustainable development studies, and (2) the accessibility of these publications to a wide international audience. The reason for choosing these two principles was to avoid articles focused only on specific areas. This study examined 33 key publications.

Using the Nvivo software package (QSR international, Burlington, MA, USA), this study conducted content analysis and established elements representing the four sustainable development dimensions. First, open coding was performed to select 26 indicators and 56 initial sentences that contained relevant factors related to the sustainable development of MIPs. Next, the 26 indicators and 56 initial sentences were divided into the 4 identified dimensions based on their contents. Finally, through summarization and comparison, the indicators or sentences with the same or similar meanings were combined to form a final indicator list containing 22 elements.

### 2.2. Stage 2

#### 2.2.1. Expert Interviews

This stage included semi-structured interviews with experts. It improved the preliminary list of MIP risks identified in Stage 1, and ensured that the risk factors were reasonable and understandable during the questionnaire survey. In total, 13 experts were invited by phone or email, and 10 experts agreed to participate in the study, as shown in Table 1. There were six participants and four scholars, almost all of whom had more than 8 years of relevant experience (Table 1). Expert interviews were conducted from 15 September to 27 October 2020, with all the interview sessions being recorded. During the expert interviews, we first surveyed the respondents’ perception of the risk factors of MIPs. All the interview questions are included in Appendix B. Based on the interview results, we checked whether the existing risk list systematically reflected the risk factors involved in MIPs. Next, the experts were asked to evaluate whether the factors on the risk list were reasonable and to check whether the wording could be understood by them.

#### 2.2.2. Questionnaire Survey

The perceptions of the respondents regarding different risk factors were based on their project experience and could be used directly for risk assessment and key risk identification. Although the attitudes (negative or passive) of the respondents caused bias between their perceptions and fact, this total perception bias of all samples was minimized by the large sample size. The perceptions of the respondents were obtained through a two-stage questionnaire: (1) relative personal information, such as years of working, educational background, the institution in the MIPs, and main responsibility; and (2) the evaluation of the probability and severity of each risk factor in the MIPs. This study used a Likert 5-point scale to rate the probability and severity—for the probability, 1, 2, 3, 4, and 5 represented rare, unlikely, possible, likely, and almost certain, respectively; for the severity, 1, 2, 3, 4, and 5 represented insignificant, minor, moderate, mega, and severe, respectively. The questionnaire content is presented in Appendix B.

From 2–28 November 2020, this study distributed a total of 231 questionnaires to relevant specialists with practical experience in MIPs. All respondents were familiar with and had participated in MIPs. After suitable filtering, 163 of the 183 received questionnaires were deemed to be effective as they had sufficient data to rank the risk factors. The response rate was 79.22%, and the effective rate was 70.56%. The number of samples met the requirement that the number of samples need to be more five times of the number of elements. Table 2 shows the details of the relative personal information. The respondents comprised contractors (41.11%), scientific research institutions (15.95%), government (19.63%), and engineering consultancies (23.31%). All the respondents had more than two years of practical experience in MIPs and a bachelor’s degree or more, indicating that they had the ability to assess each risk factor’s probability and severity.

### 2.3. Stage 3 (Fuzzy Set Theory)

The respondents’ opinions can often be subjective and vague. Many scholars have adopted the fuzzy set theory to solve this problem in risk research [35,36], as it helps to quantify the imprecise or difficult factors in respondents’ opinions [35] and determine the actual key risk factors. In this study, the fuzzy set theory was used. Its implementation was divided into the following three steps:

Step 1: Based on the probability ranking and risk factor severity from each respondent, calculate the degree to which each risk factor meets different levels of probability and degrees of severity. Moreover, consider that respondents with more working experience tend to rank each risk factor more accurately. Consequently, in this study, the respondents’ evaluation was based on their years of work. The equations are as follows:

(1) $$r_{i}=\frac{Y\times G_{i}}{Y_{\mathrm{total}}}$$

(2) $$G_{i}={\left(g_{jk}^{i}\right)}_{38\times 5}$$

(3) $$Y={\left(y_{k}^{}\right)}_{1\times 38}$$

where *Y* represents the working experience of respondents—1 = less than 2 years, 2 = 3 to 5 years, 3 = 6 to 10 years, 4 = 11 to 15 years, and 5 = more than 15 years. *Y_total_* represents the sum of all the respondents’ working experience, *j* represents the code of the respondents, *G_i_* represents the score matrix of risk factor *i* ($g_{jk=1\;or\;0}$*,* and $\overset{5}{\underset{j=1}{\sum }}g_{jk}=1$), $r_{i}$ is the member degree function of risk factor *i*, and *j* represents the level of probability and severity (*j* = 1, 2, 3, 4, 5).

Step 2: Calculate the probability, severity, and impact of risk factor *i* based on Equations (4)–(6).

(4) $$P_{i}=\sum_{j=1}^{5}\left(j\times r_{ij}^{P}\right)$$

(5) $$S_{i}=\sum_{j=1}^{5}\left(j\times r_{ij}^{S}\right)$$

(6) $$I_{i}=\sqrt{P_{i}\times S_{i}}$$

where *P_i_* represents the probability of the risk factors, *S_i_* represents the severity of the risk factors, and *I_i_* represents the impact of the risk factors. The value range of the results is [1,5].

Step 3: Evaluate the risk impact of the different dimension. To calculate the risk impact of the different dimension, the weight of each risk factor within the dimension is first calculated based on Equations (7) and (8).

(7) $$W_{i}^{t,P}=\frac{P_{i}}{\sum_{i=1}^{m}P_{i}}$$

(8) $$W_{i}^{t,S}=\frac{S_{i}}{\sum_{i=1}^{m}S_{i}}$$

where $W_{i}^{t,P}$ represents the weight of probability of risk factor *i* within dimension *t*, $W_{i}^{t,S}$ represents the weight of severity probability of risk factor *i* within dimension *t*, and *m* represents the number of risk factors within the dimension.

For dimension *D*, the calculation of its impact requires the product of the weight and membership degree of each risk factor within the dimension. Therefore, based on Equations (9) and (10), a subordinating degree function on the local probability and the local severity of each are established:

(9) $${\left(D_{t}^{P}\right)}_{1\times 5}=(d_{1}^{P},d_{t}^{P},d_{t}^{P},d_{t}^{P},d_{t}^{P}={\left(W_{}^{P}\right)}_{1\times m}\times {\left(R_{}^{p}\right)}_{m\times 5}$$

(10) $${\left(D_{t}^{S}\right)}_{1\times 5}=(d_{1}^{S},d_{t}^{S},d_{t}^{S},d_{t}^{S},d_{t}^{I}={\left(W_{}^{S}\right)}_{1\times m}\times {\left(R_{}^{S}\right)}_{m\times 5}$$

Based on the results of the above formula, we calculated the probability value, severity value, and impact value of each dimension risk in Equations (11)–(13), respectively:

(11) $$p_{t}=\sum_{j=1}^{5}\left(j\times d_{ij}^{P}\right)$$

(12) $$S_{t}=\sum_{j=1}^{5}\left(j\times d_{ij}^{S}\right)$$

(13) $$I_{t}=\sqrt{P_{t}\times S_{t}}$$

## 3. Results and Discussion

### 3.1. Mega Infrastructure Project (MIP) Risk Identification from Extended Sustainable Development Perspective

#### 3.1.1. Sustainable Development of MIPs

After a thorough content analysis of the 33 key publications, this study summarizes 22 elements (Table 3). These are generic elements that can provide a reference to all types of MIP. The economic sustainable development of MIPs requires the minimization of the direct costs of the MIPs themselves [27,37], the indirect costs of social and environmental governance [38,39], and the maintenance and enhancement of local economies [40,41], to achieve a positive long-term economic impact. The environmental sustainable development of MIPs requires the best use of resources (for example, water, energy, and land use) [28,42,43,44,45,46], minimum waste discharge (such as gas emissions, wastewater discharge, solid waste, light, and noise) [41,47,48,49], reduced damage to the natural landscape [50,51], maintenance of the biodiversity of the natural ecological system, ecological balance, and protection of the earth as far as possible [29,52]. The social sustainable development of MIPs requires the protection of regional culture [53,54], concern for the safety and health of employees and the public [29,55], compatibility with citizens’ ideals, encouragement of social integration [20,39,40,52] guaranteed social equality [56,57], and provision of a public service that improves the quality of life of all segments of the population [38,55,58,59]. The coordination of sustainable development of the MIPs was used to support the “three pillars,” wherein the MIPs project team comprised multidisciplinary (economic, social, and environmental) professionals [29,60]; furthermore, contracts, regulations, and policies that include sustainable development were formulated [37,60], and a comprehensive project lifecycle monitoring and maintenance management system was established [27,61]. Consequently, a complete sustainability management system [30,62] was formed, which allowed all the stakeholders to participate in project decision-making [29,63]; this presented a reliable atmosphere for the sustainable development of the MIP, and ensured that the interests of other stakeholders were not affected because of the interests of one party. Therefore, the sustainable development of MIPs should integrate economic, environmental, social, and coordination dimensions at the same time. In other words, the sustainable development of MIPs should achieve the optimization of economic development, environmental harmony, and social stability under coordinated management without damaging the development of future generations and achieving the goals of the project itself. In this study, the definition of sustainable development of the MIPs is different from that of the existing studies; the latter only highlights the three dimensions, but the former also emphasizes the coordinated management of the three dimensions, which is also a novel finding of this study.

#### 3.1.2. Risk Factor Identification

Based on the conceptual model shown in Figure 2, and combing with key elements of sustainable development (i.e., EC1–CO5 in Table 1), by literature analyzing the impact on the sustainable development during the construction and operation of MIPs. The possible risk factors in the life cycle of MIP were comprehensively identified from the economic dimension (ECRFs), environmental dimension (ENRFs), social dimension (SORFs), and coordination dimension (CORFs). In addition, in order to develop a generic risk list for MIPs, this study only identifies the common risk factors and does not consider the unique risk factors arising from specific types of major infrastructure project. The unique risk factors include but not limited to radiation caused by nuclear power plant, water-borne diseases or water eutrophication in water treatment infrastructure, improper energy storage in renewable energy infrastructure, and pedestrian casualties in road infrastructure, etc., which do not occur to all kinds of MIPs. The preliminary risk list is presented in the Table A1 (Appendix A).

In the processing of expert interviews, all experts agreed that MIPs may lead the agglomeration or loss of industries, causing changes in industry spatial arrangements. Experts from government departments also mentioned that as publicly funded projects, MIPs need to serve the interests of the majority and satisfy national or local legislation. Therefore, two risk factors were added to the preliminary risk factor framework (“Negative impact on industry spatial arrangement” and “Failure to satisfy national or local legislation”). Furthermore, the interview results indicated that radiation was not a common problem for most types of MIP; as a result, the “Control of radiation” risk factor was removed in this study. Finally, according commonly used engineering terminology, the experts suggested changing “Labor, materials, and equipment cost overruns” to “Construction and installation cost overruns” and pointed out that the diversity of members’ academic majors only represents one of aspect of team diversity, thus “employee ability” would be a more appropriate term. The final risk list is shown in Table 4.

##### Risk Factors in the Economic Dimension

This dimension focuses on risk factors in the economic dimension to find the risk factors that affect the economic sustainability of MIPs. Specifically, the economy comprises two levels: project level and regional level. The project level includes the direct and indirect costs of the project, and the regional level includes the impact of the MIP on the local economy. During the construction and operational phase of the MIP, many risk factors—such as construction and installation cost overruns (ECRF1), construction delays (ECRF2), poor project quality (ECRF3), and operational and maintenance cost overruns (ECRF4) [65]—affect the direct cost (EC1) and directly lead to project budget overruns. The high indirect costs (EC2) of a project are mainly related to construction waste disposal cost overruns (ECRF5), land acquisition and relocation of migrants’ cost overruns (ECRF6), and ecosystem restoration cost overruns (ECRF7) [27], which further increase public and government expenditure on the MIP.

At a regional level, local governments often hope to obtain additional financial income or reduce the financial expenditure of related services through the project, but incorrect demand forecasts (ECRF8) and weak project debt paying abilities (ECRF9) make it difficult for local governments to obtain benefits from the project. They may even need to pay more for subsidies [66]. Conversely, MIPs need to serve local economic development (EC3), but the establishment of MIPs often requires a great deal of local government investment, and a government borrowing too much can lead to reduced investment in other industries, thereby having a negative impact on the local industrial structure (ECRF11) and industrial spatial layout (ECRF12), making it hard to promote the local economy (ECRF13), and perhaps even causing the devaluation of local economic development and residents’ assets (ECRF13) [27].

##### Risk Factors in the Environmental Dimension

This dimension focuses on risk factors in the environmental dimension. MIPs have immense scale and strong production capacity, an enormous impact on resource consumption, environmental pollution, and ecological protection. The construction and operation of MIPs consumes a huge quantity of natural resources. Unreasonable project planning, low management levels, and weak team sustainability awareness often leads to the excessive consumption of resources (ENRF5, ENRF6, ENRF7, ENRF9, ENRF10, ENRF11, ENRF12)—such as water (EN2), energy (EN3), materials (EN4), and land (EN5) [38,45]. Various types of pollution, including air, water, soil, light, and noise pollution (ENRF1, ENRF2, ENRF3, ENRF4, and ENRF13, respectively), may be generated at a construction site, which can significantly impact the local natural environment (EN6) [50]. Construction activities causing air pollution (ENRF1) include land clearance, diesel engine operation, demolition, combustion, and the use of toxic substances. Meanwhile, surface water runoff and groundwater near the construction site may be polluted by various materials used in the MIP (ENRF4). The soil at a construction site can become salinized and marshy due to construction activities (ENRF13). A large amount of construction waste and waste residue (ENRF8) is generated during the construction process itself, and the construction site produces an excessive amount of noise (ENRF2), mainly from vehicles, heavy equipment, and machinery. Users may also cause air, water, soil, light, and noise pollution during the operational and maintenance phase. If an MIP is not properly planned and designed, it may damage the local natural landscape heritage (ENRF15) owing to insufficient integration of the local natural environment (ENRF14), cause geological disasters (ENRF16), and lead to a less livable environment for residents (EN1) [5,48]. MIPs can easily disrupt ecosystems (EN7), including the destruction of biodiversity (ENRF17), the disruption of ecological balance (ENRF18), the decimation of surface vegetation (ENRF19), and the obstruction of animal migration routes (ENRF20) [52].

##### Risk Factors in the Social Dimension

This dimension focuses on risk factors from the social dimension. First, MIPs should fully respect local cultural traditions (SO1). Local culture (SORF2) not being taken into account during the design process, damage to the original local historic sites during construction (SORF1), and damage to the livable environment during operation (SORF3) clearly reduce the social value of the project [47,53]. Second, people’s lives and the safety of property (SO2) are always the most important aspects in a project—casualties during construction and operation (SORF4), employees’ exposure to occupational disease (SORF5), threats to local residents’ health (SORF7), and personal property (SORF6) [5] result in reduced social sustainability and may even cause a project to be terminated. At the same time, as a project in which is invested national capital, an MIP has the responsibility to public welfare and should support all members of society to ensure fairness (SO3), participation (SO5), and utilization (SO6). However, because of poor design and planning, access to these rights is often blocked—by means of opaque project information (SORF15), lack of consideration for disabled personnel (SORF9), and gender (SORF10)—meaning users and residents may not enjoy public resources (SORF17) or have sufficient facilities (SORF18) [55,58]. Moreover, land expropriation is also a very sensitive issue with MIPs as it not only has an economic impact but also affects the employment and quality of life of residents (SORF19 and SORF20), widening the gap between the local rich and poor (SORF8) [63]. In addition, MIPs involve multiple interest groups, and so reducing inter-group conflicts and increasing social integration (SO4) is a top priority. Conflicts between the public (SORF11 and SORF12), the construction party and the public (SORF13), and the government and the public (SORF14) may be present [39]. Finally, as the leader of a MIP, the government has the right to supervise and intervene in projects. Opportunistic decision making (SORF21), bribery and corruption (SORF22), and excessive administrative intervention (SORF23) violate moral principles (SO6) and may lead to an increase in project costs and the non-cooperation of stakeholder groups [55,59].

##### Risk Factors in the Coordination Dimension

This dimension focuses on risk factors from the coordination dimension. MIPs require a system engineering multidisciplinary approach. Therefore, to fully cope with the risk factors in MIPs, a project needs a multidisciplinary professional team (CO1). When the professional knowledge of team personnel is not complementary (COEF1), lacks experience or professional ability (COEF2), and exhibits lower sustainable awareness (CORF4), it can lead to a comprehensive review of project risk factors, putting limitations on decisions [27,63]. There are often conflicts (CORF3) between schemes of different disciplines, such as environmental and economic goals. The team may find it difficult to balance these conflicts, making it difficult for the project to develop a satisfactory scheme. Significantly, the contract lies at the heart of project construction. In the signing phase of the MIP contract, unclear sustainable development-oriented objectives (CORF5), the disobeying of national and local government laws and regulations (CORF8), and the lack of establishment of reasonable responsibility sharing (CORF6) and resource allocation mechanisms (CORF7) may directly cause a project to be interrupted or even not started [46,48]. Good project decisions (CO3) are conducive to the smooth implementation of a project. In the decision-making phase of an MIP, opaque project information (CORF11), the non-convening of multi-stakeholder participation (CORF9), and the failure to comprehensively consider the interests of all stakeholders (CORF10) may cause conflicts and the breakdown of project cooperation [27,63]. In the project management process of the MIP life cycle, management methods that fail to support information sharing (CORF15) and internal communication (CORF14), the lack of a sustainable organizational culture (CORF13), and project management plan without sustainable principles (CORF11) will fundamentally result in a lack of safeguards to achieve the sustainable development of the MIP [29,50]. In this process, the monitoring system of an MIP requires timely feedback on the completion of targets. Failure to do so may lead to the inability to ensure timely error correction, which is inconsistent with sustainable development—that is, the supervision system lacks a good organizational structure (CORF18), the main body of maintenance (CORF16), and the execution process (CORF17) are not clear, and there is no auxiliary support of the information system (CORF19) [46].

### 3.2. Key Risk Factors Identification

#### 3.2.1. Questionnaire Data Test

To guarantee the validity and reliability of the data, this study measures the Cronbach’s alpha index of the data from questionnaires. In this study, the alpha values of probability and severity were 0.842 and 0.775, respectively. Research generally requires two values greater than 0.7 [32], which indicates that the global data exhibit good reliability and validity to support the research target.

Kendall’s coefficient of concordance (W) is usually tested for the consistency of the ranking for risk factors by the respondents. In this study, the Kendall W-value and P-values for probability and severity were 0.238, 0.191, 0.000, and 0.000, respectively. According to [67], a smaller Kendall W-value indicates lower data consistency. Although this study has a low Kendall W-value, we believe there is little difference in the ranking of risk factors by the respondents since the *p*-value was less than 0.05. Furthermore, this study ranks the risk factors according to the overall simplicity.

#### 3.2.2. Rank of the Overall Risk Factors

**Figure 3:**
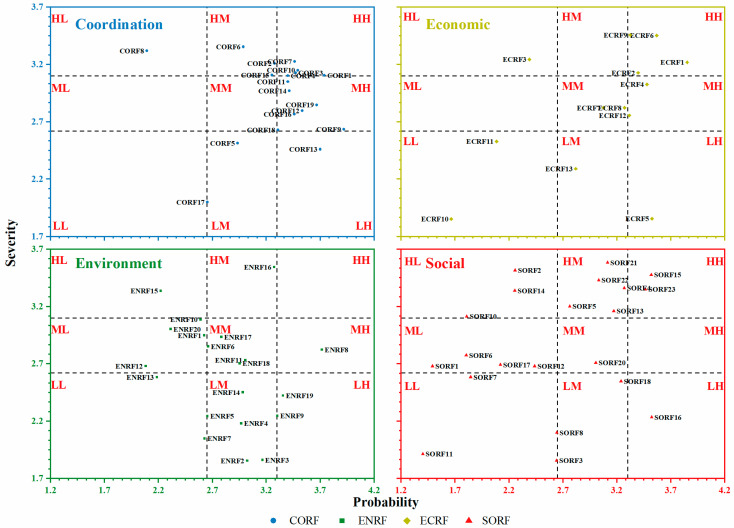
The probability and severity of overall risk factors. **Note:** CORF: coordination risk factor; ENRF: environmental risk factor; ECRF: economic risk actor; SORF: social risk factor.

The impact of risk factors is the product of probability and severity [68], but risk factors with the same impact may be completely different due to the huge difference in probability and severity [69]—such as the gray rhino risk factor (high probability and low severity) [70] and black swan risk factor (low probability and high severity) [71]. Dealing with these risk factors requires different risk response strategies. In view of this, this study analyzes and presents the results in two forms: (1) The risk factor impact ranking results, including the ranking of overall risk factors and ranking within the dimension (Table 5); (2) the probability and severity results. In this section, we use a rectangular coordinate system with the probability as the *X*-axis and the severity as the *Y*-axis, and visualize all the risk factors of the MIP (Figure 3). Based on the probability and severity, all risk factors are divided into three parts with the same number and classified into nine categories: high probability and high severity (HH), high probability and mid severity (HM), high probability and low severity (HL), mid probability and high severity (MH), mid probability and mid severity (MM), mid probability and low severity (ML), low probability and high severity (LH), low probability and mid severity (LM), and low probability and low severity (LL).

Based on the results, the distribution range of probability was (1.40, 3.92), which showed that the range of probability was from low to high. The distribution range of severity was (1.53, 3.58), indicating that the risk severity ranged from low to high. The values of the risks imparted were (1.63, 3.52)—representing risk factors—shown to be low to high, indicating that the risk list was reliable and contained important and general risk factors at the same time.

The top 10 risk factors were, in decreasing order, ECRF1, ECRF6, SORF15, CORF1, SORF23, ENRF16, ECRF9, CORF7, SORF21, and CORF10, consisting of seven high-probability risk factors and nine high-severity risk factors. The economic, social, and coordination dimensions each account for three risk factors, while the environmental dimension accounts for one risk factor. From the perspective of probability and severity distribution (Figure 3), most of the economic risk factors were located in the MH and HH areas; the social dimension risk factors were mainly distributed in the HM and ML areas; the environmental dimension risk factors were mainly distributed in the medium-impact areas, such as ML, MM, and LM; and the coordination risk factors were primarily located in the high-probability and high-severity areas (HH, MH, HM). In other words, most economic risk factors exhibit high probability, social risk factors produce high losses, environmental risk factors possess intermediate probability and severity, and coordination risk factors occur frequently. These findings were consistent with observations in previous studies [9,72].

For the impact of dimension risk, this study mainly considered the weighted score of the dimension and the average ranking of risk factors within the dimension (AR). It can be seen that CORF (3.157; AR: 25.00) > ECRF (3.147; AR: 32.23) > SORF (2.806; AR: 45.22) > ENRF (2.794; AR: 45.80).

On the one hand, this result supports the importance and necessity of introducing the coordination dimension into the risk management of MIP in this study. The decision making, planning, management, and coordination of MIPs is a process of multi-stakeholder interaction [27]. However, the application of management tools in practice has little consideration for the balance of society, economy, and environment, which results in the aggravation and escalation of multi-stakeholder conflicts and difficulties in achieving sustainable development. Stakeholders believe that the good coordination of risk factors between economic, social, and environmental dimensions has an important impact on the sustainable success of MIPs [27,29]. Coordinated management is an important driving force and an effective strategy for balancing the three dimensions [63]. On the other hand, environmental dimension and social dimension risks were lower than the economic dimension risk. This is because MIP stakeholders are not fully aware of sustainable development, and pay more attention to the cost, schedule, and quality at the project level in practice [27]. Increasing attention to the risk factors in the environmental and social dimensions needs to integrate the needs of all stakeholders to help formulate supportive regulations and incentives by the authorities.

#### 3.2.3. Rank of Economic Risk Factors

It can be seen from the table that the five most important risk factors in the economic dimension were ECRF1, ECRF6, ECRF9, ECRF2, and ECRF4.

The probability of ECRF1 was 3.219, the severity was 3.851, and the impact was 3.520. Construction and installation costs are one of the most important costs of MIPs, that is, inadequate surveys, improper design, rising material prices, and rework caused by quality problems—and can lead to an increase in costs. ECRF6 had the highest severity (3.453), and its occurrence frequency (3.568) was also in the high probability area, which indicates that resettlement remains a top priority in the implementation of MIPs. Unreasonable land expropriation and demolition work may lead to a substantial increase in the acquisition cost of construction land and antagonism from residents [57]. The operational and maintenance aspects of MIPs are relatively complex, and the operational costs are unstable and difficult to predict. The probability of ECRF4 was 3.478, the severity was 3.027, and the impact was 3.245. Operational cost overruns are related to operators and external factors [32]. Operators may submit inaccurate estimates at the bidding stage, exaggerate the funds saved for the government, and underestimate the demand of consumers, which can lead to weak operational cost controls and obstacles to the success of the service. In addition, drastic changes in the operating economic environment, such as exchange rate fluctuations, inflation, and economic crises, are beyond the control of operators, but will further aggravate operational cost overruns. Operating cost overruns lead to reduced profits, increased service costs, and poor service quality. Consequently, MIPs must focus on effective cost control strategies, including land acquisition and resettling costs, construction, and installation costs, and operational costs—reducing costs and improving benefits requires effective cost management to realize the sustainable development.

The probability of ECRF9 was 3.324, the severity was 3.457, and the impact was 3.390. Project solvency is a concern for both the government and contractors [5]. For the government, investment in MIPs takes up a large portion of government expenditure. If a project fails to recover its funding, it will have a negative impact on the local economy. For contractors, the solvency of MIPs may affect the government’s ability to pay, which will lead to a series of consequences such as insufficient cashflow of companies, the inability to settle the resulting debts, and late loan settlement, resulting in increased interest rates. Weak project debt paying ability is generally related to insufficient research in the early stages of an MIP or incorrect demand forecasts [5]. The difficulties of demand forecasting are complex—such as the uncertainty in population change, the emergence of substitution projects, and the progress of the times—and so the best method may be to predict the average values [73].

The probability of ECRF2 was 3.396, the severity was 3.127, and the impact was 3.259. Construction delays will cause a project to exceed its expected duration and are associated with significant cost overruns which may lead to claims. MIPs have a long life cycle and involve many businesses, and schedule delays may occur at each critical stage. MIPs are often more complex than ordinary projects in obtaining planning and land-use rights, requiring approval not only at the national level but also down to the local government level. Moreover, MIPs have many layers which result in more complex communication and coordination [2]. The project duration may be seriously affected because of the weak coordination ability of construction companies. Therefore, before the implementation of an MIP, a good organizational structure should be built to improve the efficiency of organizational communication, and prior approval work should be done well. During the execution of projects, the project implementation should be checked regularly, and the reasons for errors should be identified and corrected in good time.

#### 3.2.4. Rank of the Environmental Risk Factors

From the table, we can see that the five most important risk factors in the environmental dimension were ENRF16, ENRF, and ENRF11, ENRF19 and ENRF17.

The probability of ENRF16 was 3.274, the severity was 3.543, and the impact was 3.406. Geological disasters caused by MIPs include landslides, collapses, side slopes, and water and soil erosion. They are mainly caused by incomplete construction reconnaissance and improper construction measures [74]. These geological disasters may affect the normal lives of local residents and be the cause of robust public protests. Therefore, in the early stages of MIP construction, it is necessary to: strengthen geological survey and evaluation work; integrate the geological data from the MIP survey, design, and construction process; establish a geological data sharing mechanism; and provide information and technical support for the planning, site selection, construction, and operation of the MIP.

The probability of ENRF8 was 3.716, the severity was 2.822, and the impact was 3.238, making it a high probability risk and mid severity risk. The construction and operation of MIPs consumes a great deal of resources, and pollution has become a non-negligible problem [47]. Many construction waste pollution problems can be attributed to the improper disposal of construction waste [75], which causes other environmental risks such as water, soil, and air pollution, and which has a huge impact on the local environment. Therefore, the use of resources in an MIP should be accurately calculated to prevent excessive consumption, the waste generated during construction and operation should be professionally treated, and attention should be paid to the use of recyclable materials.

The probability of ENRF11 was 3.006, the severity was 2.732, and the impact was 2.866, ranking third. MIPs are large in scale and often occupy a large expanse of non-construction land, involving green space, agricultural land, and animal habitat [62]. Occupying such large expanses of non-construction land not only affects the income of local residents but, more importantly, also damages the local natural environment and ecology. Therefore, MIP construction should evaluate the rational use of land, strengthen the land-saving system, and adopt land-saving measures at all stages of development.

The probability, severity, and impact of ENRF19 were 3.356, 2.424 and 2.852 respectively. Damage to surface vegetation is a very common phenomenon in MIP implementation [62]. Usage of construction land and temporary sites usually requires the eradication of surface vegetation, which can also induce natural disasters such as soil erosion and landslides, and cause greater environmental losses, having an enormous impact on the living environment of local residents. Therefore, during MIP construction and operation, ecological restoration measures should be undertaken on the basis of employing natural processes to restore vegetation and soil, ensure a certain vegetation coverage, and maintain the self-sustaining ability of the ecosystem.

The probability of ENRF17 was 2.783, the severity was 2.934, and the impact was 2.857. Due to the encroachment on animal habitats and destruction of surface vegetation being ranked top, this risk ranking result was confirmed—the destruction of biodiversity by a project will further lead to the loss of ecological balance [63]. Therefore, MIP construction should take measures to protect important habitats of animals and plants, such as defining planning corridors, constructing biological channels, providing multiporous habitats, etc., to maintain ecological diversity.

#### 3.2.5. Rank of the Social Risk Factors

It can be seen that the five most important risk factors in the social dimension were SORF15, SORF23, SORF21, SORF22, and SORF4.

The probability of SORF15 was 3.518, the severity was 3.477, and the impact was 3.497. SORF15 was ranked third in the overall ranking and first in the social dimension. The ranking confirmed that the SORF15 was the most important social risk identified by the surveyed experts, which also happens to be a social risk that has been studied more. Opaque project information reduces the public’s trust in the government and leads to a higher probability of public protest [76]. Therefore, transparency and open exchange of information during MIP implementation are of the utmost importance. Indeed, popularization of science, communication with the public through various channels, and timely response to their concerns are critical to the realization of sustainable MIP development.

The probability of SORF23 was 3.465, the severity was 3.350, and the impact was 3.407, ranking 5th in the overall risk. Considering the political impact of MIPs, political intervention at the planning, construction, and operational stages is justified [77]. Political interference refers to the government’s interference in the activities of contractors and operators, such as the risk of adjusting policies and taxes [5]. In China, laws and regulations for the construction activities of MIPs are not incomplete, and local governments can unilaterally modify some laws and policies without consultation, leading to the failure of contractors to obtain political commitment or support and who may then refuse to continue to perform their duties, leading to service interruptions and heightened social risk. To reduce such social risk factors, the government should reasonably restrain and regulate its own behavior and effectively fulfill its supervisory powers, which may be beneficial in taking full advantage of the enthusiasm of contractors, promoting the success of the project.

SORF21 refers to making decisions at the expense of others by misleading or confusing the other party with endless information to further their own interests [78]. Its probability was 3.114, the severity was 3.582, and the impact was 3.340. Zeng et al. [59] believe that in the context of strong government and weak laws and regulations, opportunistic decision-making behavior seriously affects the social responsibility performance of MIPs. In China, MIP projects are generally led by the government, so information asymmetry between the government and the contractor occurs easily, which leads to opportunistic decision making [59], and the high probability of risk reflects this reality. In addition to improving punitive mechanisms to curb opportunistic contractor behaviors, the government can also apply a subsidy mechanism to ensure that the amount of government subsidies will be greater than the any additional benefits the contractor could obtain through asymmetric information, thereby reducing the occurrence of such social risk factors.

The probability of SORF22 was 3.032, the severity was 3.430, the impact was 3.224, and the overall risk ranking was 16. The results make sense, as in the recent years, there have been many MIP scandals, particularly with regard to bribery and corruption, which often occur during the initial bidding stages of MIPs [79]. This not only leads to quality problems and safety incidents, but also to public complaints and can seriously damage the image and credibility of the government. In order to reduce the incidence of corruption, on the one hand, the active supervision of the project should be strengthened by reducing the cost of reporting and improving the benefits of reporting; on the other hand, the passive supervision of the project by the public and social media should be strengthened by improving the project information disclosure channels.

SORF4 is an important risk factor with medium probability and high loss, whose probabilities, severity, and impact were 3.267, 3.361, and 3.314, respectively. Most MIPs are built in desolate places, and poor outdoor working conditions are more likely to cause safety accidents than in other industries. Safety has always been a key concern for people [80]. It is related to casualties, resulting in unequal opportunities for people in accidents, and will trigger a strong discussion of the right to survival and development, and affect the social value of MIPs. Therefore, in order to reduce the occurrence of safety accidents, engineering safety measures should be strengthened, and worker safety awareness and knowledge should be improved.

#### 3.2.6. Rank of the Coordination Risk Factors

It can be seen from the table that the five most important risk factors in the coordination dimension were CORF1, CORF7, CORF10, CORF3, and CORF4.

CORF1 ranked first in coordination dimension, and the scores for probability, severity, and impact were 3.739, 3.105, and 3.408, respectively. MIPs are a complex multidisciplinary system. The singular ability of the team largely limits the comprehensive understanding of MIPs and cannot comprehensively consider the economic, environmental, and social goals, thereby affecting the rationality of decision making. Li et al. [81] pointed out that team members of MIPs should include talents in various fields, emphasizing comprehensive quality capabilities and field balance. Therefore, the establishment of a multi-disciplinary, multi-field management team could reduce the risk associated with insufficient management capabilities.

The rank of CORF7 confirmed that great contracts are considered to be one of the most important factors for the success of MIPs, ranking second. The probability, severity, and impact of this risk were 3.463, 3.227, and 3.343, respectively. Stakeholder coordination in MIPs largely depends on the distribution of benefits among them [82]. The investment and resource allocation clause in a contract outlines the rules and guides the behavior of stakeholders. This is a key factor in ensuring the successful implementation of MIPs. Missing or incorrect setting of contractual clauses will cause conflict. This result also reminds us that it is necessary to draft agreed investment and resource allocations and set performance targets in the contract.

CORF10 had high probability (3.493), high severity (3.150), and high impact (3.317). Because of the large number of participants and different interests in MIPs, conflicts are bound to arise [27]. For example, the government has to bear the triple responsibilities of developing the economy, maintaining social stability, and protecting the ecological environment, while contractors of MIPs mainly pursue profits [32], and the public is more concerned about whether the project poses risks with potentially negative effects on their own lives. As Ma et al. [82] pointed out, if the balance of interests of all parties is not equitable, the effect of decision-making will be greatly reduced. Therefore, it is necessary to understand the needs of all stakeholders during the decision-making stage, consider the risk tolerance, risk preference, risk perception ability and other factors of each stakeholder, and coordinate the interests of different stakeholders to achieve the most sustainable development.

CORF3 ranked fourth in the coordination dimension, with high probability (3.472), high severity (3.127), and high impact (3.295). The internal conflicts of MIPs not only originate from owners and contractors, but also include bad partnerships between different owners, owner consultants, different contractors, consultants, suppliers, and other stakeholders [83]. Because team members have different professions, their goals will be different. When management philosophy and mentality are not carried out in true partnership, it is difficult to avoid hostility between the parties even with a great contract [81]. Team conflicts will affect the harmonious atmosphere within the team, waste time and resources, affect the performance of an MIP and threaten its success. Therefore, an MIP team should adopt a common method of cooperation, establish a cooperative partnership, and actively promote effective relationship management to ensure that all members succeed.

The probability of CORF4 was 3.400, the severity was 3.102, and the impact was 3.247, ranking fifth in the coordination dimension. Given the stark lack of knowledge of sustainability practices among practitioners in the construction industry in China, this finding is reasonable [38]. MIPs attach great importance to the economic performance of the project and pay less attention to developing the social economy, building a harmonious society, and reducing the negative impact on the ecological environment. This leads to an inadequate consideration of social and environmental risk factors and can cause public resistance and polarize social opinion. The results of this study also highlight the important role of team members’ sustainability awareness. Therefore, the concept of sustainable development should be incorporated into the project risk management culture, so that all project stakeholders are aware of its purpose, driving them to exert their own subjective initiative to manage the associated risks.

## 4. Conclusions

To realize the sustainable development of MIPs and implement adequate risk control measures for them, it is necessary to have an in-depth understanding of the definition of sustainable development of MIPs, find out the actual risk factors that may be encountered in achieving the sustainable development goals, and identify key risk factors having a significant impact on the projects and requiring the attention of project stakeholders. In order to address the above issues, this study extended the definition of sustainable development of MIPs by combining the triple bottom lines with a fourth coordination dimension to propose a conceptual model for MIPs risk identification from an extended sustainable development perspective, while identifying 22 sustainable elements through a literature review and 75 risk factors for MIPs by analyzing the negative effects that lead to deviations of projects from sustainable development elements. Based on questionnaire surveys and the fuzzy set theory, the key risk factors are identified.

The main results were as follows: (1) Coordination risk dimensions had the highest impact, which indicated that the three dimensions of TBL theory must be coordinated and balanced to promote the sustainable development of the project, supporting the importance and necessity of introducing the coordination dimension into the MIP risk management process. (2) Risk factors of different dimensions represented different characteristics. Specifically, economic risk factors were highly probable, social risk factors produced high losses, the probability and severity of environmental risk factors occurred with intermediate probability and severity, and coordination risk factors had high impact. (3) The key risk factors mainly concentrated on economic, social and coordination dimensions. The 10 most important risk factors were (impact from high to low): “construction and installation cost overruns” (ECRF1), “land acquisition and resettling cost overruns” (ECRF6), “information sharing to the public” (SORF15), “non-complementary employee ability” (CORF1), “excessive government intervention” (SORF23), “causing geological hazards” (ENRF16), “weak solvency ability” (ECRF9), “inadequate investment and source sharing clauses” (CORF7), “opportunistic decision making” (SORF21), and the “difficulty of coordinating interest demand” (CORF10).

From the sustainable development perspective, this study has academic and practical implications for the risk management of MIPs. The extended definition of sustainable development of MIPs, the comprehensive and systemic sustainable development element list and the risk factor list deliver a new perspective and reference for studies on MIPs risk management. The ranking of dimension risks and risk factors weighted by respondents’ working experience is conducive to improving MIPs decision makers’ comprehension of the risk factors of MIPs (including the content, impact, probability, and severity), providing an effective reference for the establishment of a reasonable risk early warning mechanism, creating reasonable risk response strategies, strengthening coordination and administration for the achievement of MIPs economic, social and environmental goals, diminishing the negative effects on TBL goals, and boosting the sustainable performance of MIPs.

Although this study fills in some research gaps, three deficiencies should be noted. (1) The MIP risk factors identified in this study were based on the specific conditions in China. As MIPs in different countries and regions will face different requirements for sustainable development, the proposed conceptual model for risk identification and the associated key risks must be verified in the future to determine their applicability to other countries. (2) The respondents of this study were mainly participants with significant practical infrastructure or research experience, and public opinion was not considered. The public is one of the most important stakeholders in MIP development. A clear understanding of the public’s perception and attitude toward MIP risk factors from an extended sustainable development perspective is critical for coordinating contradictions between stakeholder interests and achieving sustainable development. (3) Many studies have reported that there is an interactive relationship between the risk factors, which may magnify or diminish the probability and severity of the risk; however, in this study, the aforementioned effect was not considered. The authors, therefore, intend to further explore these deficiencies in future studies to establish a risk ranking methodology that will take public perception and risk interaction into account to provide improved guidance for MIP risk management from a sustainable development perspective.

## Figures and Tables

**Table 1:** The information of interviewed experts.

ID	Experience (Years)	Role	Organization
Expert1	12	Scholar	University
Expert2	19	Project participant	Construction contractor
Expert3	15	Project participant	Construction contractor
Expert4	19	Project participant	Consultant
Expert5	20	Scholar	University
Expert6	19	Project participant	Construction contractor
Expert7	17	Scholar	University
Expert8	9	Project participant	Consultant
Expert9	11	Project participant	Construction contractor
Expert10	13	Scholar	University

**Table 2:** The personal information of respondents.

The Role of Respondents	Valid Questionnaire	Ratio	Experience	Valid Questionnaire	Ratio	Degree	Valid Questionnaire	Ratio
Contractor	67	41.11%	<2	47	28.83%	Bachelor	77	47.24%
Scholar	26	15.95%	3–5	26	15.95%	Master	54	33.13%
Government	32	19.63%	6–10	34	11.04%	Doctor	32	19.63%
Consultation enterprises	38	23.31%	11–15	38	23.31%			
			>15	18	20.86%			

**Table 3:** The key elements of sustainable development.

Dimensions	Element	Code	Sources
Economy	Direct cost	EC1	[27,37]
	Indirect cost	EC2	[38,39]
	Local economy	EC3	[40,41]
Environment	Atmosphere	EN1	[47,49]
	Water	EN2	[28,42]
	Energy	EN3	[43,46]
	Construction materials	EN4	[41,48]
	Land	EN5	[44,45]
	Landscape	EN6	[5,50]
	Ecology	EN7	[29,52]
Society	Cultural	SO1	[53,54]
	Health and safety	SO2	[29,55]
	Social equity	SO3	[56,57]
	Social integration	SO4	[29,52]
	Participation	SO5	[39,40]
	Public utility	SO6	[38,58]
	Ethical	SO7	[55,59]
Coordination	Teamwork of multidisciplinary professionals	CO1	[29,64]
	Contracts, regulations and policies	CO2	[37,64]
	Decision-making of all stakeholders	CO3	[29,63]
	Management system for sustainability	CO4	[30,62]
	Management system for lifecycle monitoring and maintenance	CO5	[27,61]

**Table 4:** The risk factors of mega infrastructure projects (MIP).

Dimension	Elements	Code	Risk Factors
Economy	Direct cost	ECRF1	Construction and installation cost overruns
		ECRF2	Construction delay
		ECRF3	Quality failures
		ECRF4	Operation and maintain cost overruns
	Indirect cost	ECRF5	Disposal of construction waste cost overruns
		ECRF6	Land acquisition and resettling cost overruns
		ECRF7	Ecological remediation cost overruns
	Local economy	ECRF8	Wrong market demand forecasts (overrate)
		ECRF9	Weak solvency ability
		ECRF10	Devaluation of residents’ assets (decrease in residents’ income)
		ECRF11	Negative impact on the local industrial structure (tourism, agriculture, etc.)
		ECRF12	Negative impact on the spatial layout of local industries
		ECRF13	Weak contribution on Local economy
Environment	Atmosphere	ENRF1	Air pollutant (greenhouse gases, toxic gases, dust)
		ENRF2	Control of noises
		ENRF3	Control of lights
	Water	ENRF4	Water pollution
		ENRF5	Overuse of water resources
	Energy	ENRF6	Excessive consumption of non-renewable energy
		ENRF7	Overuse of renewable energy
	Construction materials	ENRF8	Construction waste pollution (solid waste pollution)
		ENRF9	Overuse of construction materials
		ENRF10	Usage of not environmental-friendly construction materials
	Land	ENRF11	Occupy a lot of non-construction land (green land, agricultural land, animal habitat)
		ENRF12	Idle of developed land
		ENRF13	Soil health degradation (salinization, swamping, etc.)
	Landscape	ENRF14	Non-matching with natural environment
		ENRF15	Damage to natural heritage
	Ecology	ENRF16	Causing geological hazards (landslide, collapse, slope instability, soil erosion, etc.)
		ENRF17	Damage to biodiversity
		ENRF18	Damage to the ecological balance
		ENRF19	Damage to surface plants
		ENRF20	Obstruction on animal migration
Society	Cultural	SORF1	Damages of Cultural Heritage
		SORF2	Non-matching with local culture
		SORF3	Negative impact on quality of life
	Health and safety	SORF4	Construction safety and accidents
		SORF5	Occupational disease
		SORF6	Damage to residents’ safety (personal or property)
		SORF7	Damage to residents’ health
	Social equity	SORF8	Widen the gap between rich and poor
		SORF9	No access of the disabled
		SORF10	Sexual discrimination
	Social Integration	SORF11	Marginalization of immigrants
		SORF12	Damage to connectivity among communities
		SORF13	Discoordination between contractor and public
		SORF14	Conflict between government and public
	Participation	SORF15	Information sharing to the public (closed decision information)
		SORF16	Damage to participation of local resident
		SORF17	No access to public resources to local residents
	Public utility	SORF18	Inadequate facilities surrounding the projects
		SORF19	Negative impact on employment (unemployment, underutilization of local labor force)
		SORF20	Unreasonable resettlement
	Ethical	SORF21	Opportunism decision making
		SORF22	Bribery and corruption
		SORF23	Excessive government intervention
Coordination	Teamwork of multidisciplinary professionals	CORF1	Non-complementary employee ability
		CORF2	Inadequate experience
		CORF3	Team conflict (non-cooperation)
		CORF4	Weak sustainability awareness
	Contract, regulations and policies	CORF5	Lack of sustainable clauses in contract
		CORF6	Ambiguous responsibility and right sharing clauses
		CORF7	Inadequate investment and source sharing clauses
		CORF8	Failure to satisfy national or local legislation
	Decision-making of all stakeholders	CORF9	Decision-making of all stakeholders
		CORF10	Difficulty of coordinating interest demand
		CORF11	Weak and opaque decision-making process
	Management system for sustainability	CORF12	Ambiguous sustainable management program
		CORF13	Lack of organization culture on sustainability
		CORF14	Incomplete communication and coordination procedures
		CORF15	Information sharing to all stakeholders
	Management system for lifecycle monitoring and maintenance	CORF16	Unclear maintenance subjects of project sustainability
		CORF17	Unclear monitor system of project sustainability
		CORF18	Unclear monitor and maintenance organization of project sustainability
		CORF19	Weak monitor and maintenance platform of project sustainability

**Table 5:** The rank of risk factors.

Code	Dimension	Probability	Severity	Impact	The Rank in Overall Risks	The Rank in Dimension
ECRF1	Economic	3.851	3.219	3.520	1	1
ECRF6	Economic	3.568	3.453	3.510	2	2
SORF15	Social	3.518	3.477	3.497	3	1
CORF1	Coordination	3.739	3.105	3.408	4	1
SORF23	Social	3.465	3.350	3.407	5	2
ENRF16	Environmental	3.274	3.543	3.406	6	1
ECRF9	Economic	3.324	3.457	3.390	7	3
CORF7	Coordination	3.463	3.227	3.343	8	2
SORF21	Social	3.114	3.582	3.340	9	3
CORF10	Coordination	3.493	3.150	3.317	10	3
SORF4	Social	3.267	3.361	3.314	11	4
CORF3	Coordination	3.472	3.127	3.295	12	4
ECRF2	Economic	3.396	3.127	3.259	13	4
CORF4	Coordination	3.400	3.102	3.247	14	5
ECRF4	Economic	3.478	3.027	3.245	15	5
CORF2	Coordination	3.276	3.209	3.242	16	6
ENRF8	Environmental	3.716	2.822	3.238	17	2
CORF19	Coordination	3.667	2.848	3.232	18	7
SORF22	Social	3.032	3.430	3.224	19	5
CORF11	Coordination	3.400	3.049	3.220	20	8
CORF9	Coordination	3.918	2.635	3.213	21	9
CORF14	Coordination	3.413	2.971	3.184	22	10
CORF15	Coordination	3.253	3.107	3.179	23	11
SORF13	Social	3.171	3.160	3.165	24	6
CORF6	Coordination	2.987	3.354	3.165	25	12
CORF12	Coordination	3.533	2.797	3.143	26	13
CORF16	Coordination	3.461	2.768	3.095	27	14
ECRF8	Economic	3.272	2.822	3.039	28	6
ECRF12	Economic	3.315	2.756	3.022	29	7
CORF13	Coordination	3.699	2.461	3.017	30	15
SORF5	Social	2.762	3.201	2.974	31	7
CORF18	Coordination	3.307	2.629	2.949	32	16
ECRF7	Economic	3.076	2.822	2.946	33	8
SORF18	Social	3.238	2.549	2.873	34	8
ENRF11	Environmental	3.006	2.732	2.866	35	3
ENRF17	Environmental	2.783	2.934	2.857	36	4
SORF20	Social	3.004	2.709	2.853	37	9
ENRF19	Environmental	3.356	2.424	2.852	38	5
ENRF10	Environmental	2.592	3.086	2.828	39	6
ENRF18	Environmental	2.954	2.703	2.826	40	7
SORF2	Social	2.257	3.516	2.817	41	10
SORF16	Social	3.522	2.234	2.805	42	11
ECRF3	Economic	2.389	3.244	2.784	43	9
ENRF1	Environmental	2.625	2.947	2.782	44	8
ENRF6	Environmental	2.661	2.852	2.755	45	9
SORF14	Social	2.253	3.338	2.742	46	12
ENRF9	Environmental	3.303	2.248	2.725	47	10
ENRF15	Environmental	2.221	3.336	2.722	48	11
CORF5	Coordination	2.933	2.514	2.715	49	17
ENRF14	Environmental	2.983	2.453	2.705	50	12
ENRF20	Environmental	2.314	3.002	2.635	51	13
CORF8	Coordination	2.093	3.318	2.635	52	18
ECRF5	Economic	3.526	1.855	2.558	53	10
SORF12	Social	2.438	2.680	2.556	54	13
ENRF4	Environmental	2.968	2.182	2.545	55	14
ECRF13	Economic	2.817	2.291	2.540	56	11
ENRF5	Environmental	2.655	2.244	2.441	57	15
ENRF3	Environmental	3.164	1.861	2.427	58	16
SORF17	Social	2.120	2.691	2.389	59	14
ENRF13	Environmental	2.185	2.584	2.376	60	17
SORF10	Social	1.808	3.113	2.373	61	15
ENRF2	Environmental	3.023	1.855	2.368	62	18
ENRF12	Environmental	2.082	2.680	2.362	63	19
SORF8	Social	2.644	2.100	2.356	64	16
ENRF7	Environmental	2.627	2.049	2.320	65	20
CORF17	Coordination	2.655	2.000	2.304	66	19
ECRF11	Economic	2.084	2.529	2.296	67	12
SORF6	Social	1.804	2.773	2.237	68	17
SORF3	Social	2.640	1.857	2.214	69	18
SORF7	Social	1.844	2.584	2.183	70	19
SORF1	Social	1.491	2.680	1.999	71	20
ECRF10	Economic	1.665	1.854	1.757	72	13
SORF9	Social	1.933	1.539	1.725	73	21
SORF11	Social	1.402	1.914	1.638	74	22
SORF19	Social	1.731	1.551	1.638	75	23

## Data Availability

The data presented in this study are available on request from the corresponding author.

## Appendix A. Risk List

**Table A1:** Preliminary risk list.

Code	Risk Factors
ECRF1	Labor, materials, and equipment cost overruns
ECRF2	Construction delay
ECRF3	Quality failures
ECRF4	Operation and maintenance cost overruns
ECRF5	Disposal of construction waste cost overruns
ECRF6	Land acquisition and resettling cost overruns
ECRF7	Ecological remediation cost overruns
ECRF8	Wrong market demand forecasts (overrate)
ECRF9	Weak solvency ability
ECRF10	Devaluation of residents assets
ECRF11	Negative impact on industry structure
ECRF13	Weak contribution on Local economy
ECRF14	Underuse of water recycling
ECRF15	Control of radiation
ENRF1	Air pollutant (greenhouse gases, toxic gases, dust)
ENRF2	Control of noises
ENRF3	Control of lights
ENRF4	Water pollution
ENRF5	Excessive consumption of construction water
ENRF6	Excessive consumption of renewable energy
ENRF7	Excessive consumption of renewable energy
ENRF8	Construction waste pollution (solid waste pollution)
ENRF9	Excessive consumption of construction materials
ENRF10	Usage of not environmental-friendly construction materials
ENRF11	Usage of Land unfit for construction (green space, agricultural land and animal habitat)
ENRF12	Idle of developed land
ENRF13	Soil health degradation (salinization, swamping, etc.)
ENRF14	Non-matching with natural environment
ENRF15	Damage to natural heritage
ENRF16	Causing geological hazards (landslide, collapse, slope instability, soil erosion, etc.)
ENRF17	Damage to biodiversity
ENRF18	Damage to the ecological balance
ENRF19	Damage to surface plants
ENRF20	Obstruction on animal migration
SORF1	Damages of Cultural Heritage
SORF2	Non-matching with local culture
SORF3	Negative impact on quality of life
SORF4	Construction safety and accidents
SORF5	Occupational disease
SORF6	Damage to residents’ safety (personal or property)
SORF7	Damage to residents’ health
SORF8	Widen the gap between rich and poor
SORF9	No access of the disabled
SORF10	Sexual discrimination
SORF11	Marginalization of immigrants
SORF12	Damage to connectivity among communities
SORF13	Incoordination between contractor and public
SORF14	Conflict between government and public
SORF15	Information sharing to the public (closed decision information)
SORF16	Damage to participation of local resident
SORF17	No access to public resources to local residents
SORF18	Inadequate facilities surrounding the projects
SORF19	Negative impact on employment (unemployment, underutilization of local labor force)
SORF20	Unreasonable resettlement
SORF21	Opportunism decision making
SORF22	Bribery and corruption
SORF23	Excessive governmental intervention
CORF1	Single subject majored by members
CORF2	Inadequate experience
CORF3	Team conflict(non-cooperation)
CORF4	Weak sustainability awareness
CORF5	Lack of sustainable clauses in contract
CORF6	Ambiguous responsibility and right sharing clauses
CORF7	Inadequate investment and source sharing clauses
CORF8	Decision-making of all stakeholders
CORF9	Difficulty of coordinating interest demand
CORF10	Weak and opaque decision-making process
CORF11	Ambiguous sustainable management program
CORF12	Lack of organization culture on sustainability
CORF13	Incomplete communication and coordination procedures
CORF14	Information sharing among all stakeholders
CORF15	Unclear maintenance subjects of project sustainability
CORF16	Unclear monitor system of project sustainability
CORF17	Unclear monitor and maintenance organization of project sustainability
CORF18	Weak monitoring and maintenance platform of project sustainability

## Appendix B. Interview Questions and Questionnaire Contents

### Appendix B.1. Interview Questions

#### Appendix B.1.1. Phase 1 Interview

1. What is your role in the project?
2. How to understand the sustainability of mega infrastructure projects?
3. What is the impact of sustainability on risk management of mega infrastructure projects?
4. What risks (economic dimension) need special attention in the implementation of major infrastructure projects?
5. What risks (environmental dimension) need special attention in the implementation of major infrastructure projects?
6. What risks (social dimension) need special attention in the implementation of major infrastructure projects?
7. How do project team coordinate the three dimensions of mega infrastructure projects or what capabilities do project team need to have?

#### Appendix B.1.2. Phase 2 Interview

8. Does the risk list comprehensively cover risk factors of mega infrastructures projects? Whether to add or delete?
9. Is the wording of all risk factors understandable?

### Appendix B.2. Questionnaire Contents

#### Appendix B.2.1. First Part: Personal Information

1. Please list the Mega infrastructure projects you have been involved in?
2. The role of your organization in Mega infrastructure projects
3. In the construction of major infrastructure projects, what are the project stages your organization participates in?
4. Your years of working?
5. Your highest educational attainment?
6. What’s your major?

#### Appendix B.2.2. Second Part: Questionnaire Guide

The questionnaire aims to investigate the probability and the Severity of risk factors in the economic, environmental, social and project coordination dimensions of the sustainable development of MIPs. The following criteria are adopted respectively.

Select the risk factor probability, and the following scoring rules are used for the probability of risk occurrence:

**Table A2:** Risk frequency scoring rules.

Score	Definition
5	Very frequent
4	Frequent
3	Ordinary
2	Rare
1	Hardly

Choose the risk severity in the economic environmental, social, and project coordination, and the following scoring rules are used for the probability of risk occurrence:

**Table A3:** Risk severity scoring rules.

Score	Definition
5	Most severe
4	Very severe
3	Severe
2	Possibly severe
1	Can be ignored

#### Appendix B.2.3. Third Part: Risk factor Assessment of Mega Infrastructure Projects

**Table A4:** Risk factor assessment.

Dimension	Code	Risk Factors	Probability	Severity
Economy	ECRF1	Construction and installation cost overruns		
	ECRF2	Construction delay		
	ECRF3	Quality failures		
	ECRF4	Operation and maintain cost overruns		
	ECRF5	Disposal of construction waste cost overruns		
	ECRF6	Land acquisition and resettling cost overruns		
	ECRF7	Ecological remediation cost overruns		
	ECRF8	Wrong market demand forecasts (overrate)		
	ECRF9	Weak solvency ability		
	ECRF10	Devaluation of residents assets		
	ECRF11	Negative impact on industry structure		
	ECRF12	Negative impact on industry spatial arrangement		
	ECRF13	Weak contribution on local economy		
	Supplement			
Environment	ENRF1	Air pollutant (greenhouse gases, toxic gases, dust)		
	ENRF2	Control of noises		
	ENRF3	Control of lights		
	ENRF4	Water pollution		
	ENRF5	Excessive consumption of construction water		
	ENRF6	Excessive consumption of renewable energy		
	ENRF7	Excessive consumption of renewable energy		
	ENRF8	Construction waste pollution (solid waste pollution)		
	ENRF9	Excessive consumption of construction materials		
	ENRF10	Usage of not environmental-friendly construction materials		
	ENRF11	Usage of Land unfit for construction (green space, agricultural land and animal habitat)		
	ENRF12	Idle of developed land		
	ENRF13	Soil health degradation (salinization, swamping, etc.)		
	ENRF14	Non-matching with natural environment		
	ENRF15	Damage to natural heritage		
	ENRF16	Causing geological hazards (landslide, collapse, slope instability, soil erosion, etc.)		
	ENRF17	Damage to biodiversity		
	ENRF18	Damage to the ecological balance		
	ENRF19	Damage to surface plants		
	ENRF20	Obstruction on animal migration		
	Supplement			
Social	SORF1	Damages of Cultural Heritage		
	SORF2	Non-matching with local culture		
	SORF3	Negative impact on quality of life		
	SORF4	Construction safety and accidents		
	SORF5	Occupational disease		
	SORF6	Damage to residents’ safety (personal or property)		
	SORF7	Damage to residents’ health		
	SORF8	Widen the gap between rich and poor		
	SORF9	No access of the disabled		
	SORF10	Sexual discrimination		
	SORF11	Marginalization of immigrants		
	SORF12	Damage to connectivity among communities		
	SORF13	Incoordination between contractor and public		
	SORF14	Conflict between government and public		
	SORF15	Information sharing to the public (closed decision information)		
	SORF16	Damage to participation of local resident		
	SORF17	No access to public resources to local residents		
	SORF18	Inadequate facilities surrounding the projects		
	SORF19	Negative impact on employment (unemployment, underutilization of local labor force)		
	SORF20	Unreasonable resettlement		
	SORF21	Opportunism decision making		
	SORF22	Bribery and corruption		
	SORF23	Excessive governmental intervention		
	Supplement			
Coordination	CORF1	Non-complementary employee ability		
	CORF2	Inadequate experience		
	CORF3	Team conflict(non-cooperation)		
	CORF4	Weak sustainability awareness		
	CORF5	Lack of sustainable clauses in contract		
	CORF6	Ambiguous responsibility and right sharing clauses		
	CORF7	Inadequate investment and source sharing clauses		
	CORF8	Unsatisfying national or local legislation		
	CORF9	Decision-making of all stakeholders		
	CORF10	Difficulty of coordinating interest demand		
	CORF11	Weak and opaque decision-making process		
	CORF12	Ambiguous sustainable management program		
	CORF13	Lack of organization culture on sustainability		
	CORF14	Incomplete communication and coordination procedures		
	CORF15	Information sharing to all stakeholders		
	CORF16	Unclear maintenance subjects of project sustainability		
	CORF17	Unclear monitor system of project sustainability		
	CORF18	Unclear monitor and maintenance organization of project sustainability		
	CORF19	Weak monitor and maintenance platform of project sustainability		
	Supplement			

Note: If any risk factors need to be added, please add them in the supplement of Table A4.
